# Myeloma‐Associated Sporadic Late‐Onset Nemaline Myopathy, Successfully Treated with Daratumumab Based Induction and Autologous Stem Cell Transplantation: A Case Report

**DOI:** 10.1002/jha2.70390

**Published:** 2026-09-15

**Authors:** Kenji Moriwaki, Yawara Kawano, Makoto Nakajima, Nao Nishimura, Mitsuharu Ueda, Jun‐ichirou Yasunaga

**Affiliations:** ^1^ Department of Hematology Rheumatology and Infectious Diseases Graduate School of Medical Sciences Kumamoto University Kumamoto Japan; ^2^ Department of Neurology Graduate School of Medical Sciences Kumamoto University Kumamoto Japan

**Keywords:** ASCT, case report, daratumumab, myeloma, SLONM

## Abstract

Sporadic late‐onset nemaline myopathy (SLONM) is a rare, acquired myopathy often associated with monoclonal gammopathy. We report a 48‐year‐old man presenting with progressive proximal and truncal muscle weakness in whom SLONM associated with smoldering myeloma was highly suspected. He received daratumumab, lenalidomide, and dexamethasone followed by high‐dose melphalan with autologous stem cell transplantation and posttransplant therapy. Treatment resulted in marked improvement in muscle strength and performance status. The patient achieved minimal residual disease negativity and has remained relapse‐free for 5 years. This case highlights the potential efficacy of daratumumab‐based therapy followed by transplantation in SLONM associated with plasma cell dyscrasia.

Trial Registration: The authors have confirmed clinical trial registration is not needed for this submission.

## 1 Introduction

Sporadic late‐onset nemaline myopathy (SLONM), first reported in 1966 [1, 2], is a rare disease with fewer than 200 cases documented to date [3]. It is a myopathic disorder primarily characterized by muscle weakness and muscle atrophy. Diagnosis is based on pathological findings, including the presence of nemaline rods on Gomori trichrome staining and various myopathic changes, in combination with the clinical course [4].

SLONM typically presents predominant proximal muscle weakness and often follows a progressive course. Muscle weakness is especially prominent in the proximal limb and trunk muscles, and respiratory muscle involvement may lead to respiratory failure. SLONM is frequently associated with monoclonal gammopathy of undetermined significance (MGUS), in which cases the disease course tends to be more aggressive and the prognosis poorer [3, 5, 6].

Here, we report a case of SLONM complicated by smoldering myeloma that showed a favorable clinical course with daratumumab, lenalidomide, and dexamethasone (DLd) therapy, followed by autologous stem cell transplantation (ASCT), resulting in remission and minimal residual disease (MRD) negativity.

## 2 Case Presentation

A 48‐year‐old man with a medical history of Type 2 diabetes mellitus and hypertension developed slowly progressive fatigue and proximal muscle weakness in all four limbs over 4 years. The patient was referred to in the Department of Neurology at Kumamoto University Hospital for evaluation of myopathy.

Physical examination revealed dropped head posture, skin hyperpigmentation, and mild bilateral lower leg edema. No dermatological findings suggestive of dermatomyositis, such as Gottron's papules or heliotrope rash, were observed. Manual muscle testing demonstrated weaknesses of the cervical, truncal, and proximal muscles of both upper and lower extremities, leading to low performance status (ECOG: PS‐3).

**FIGURE 1:**
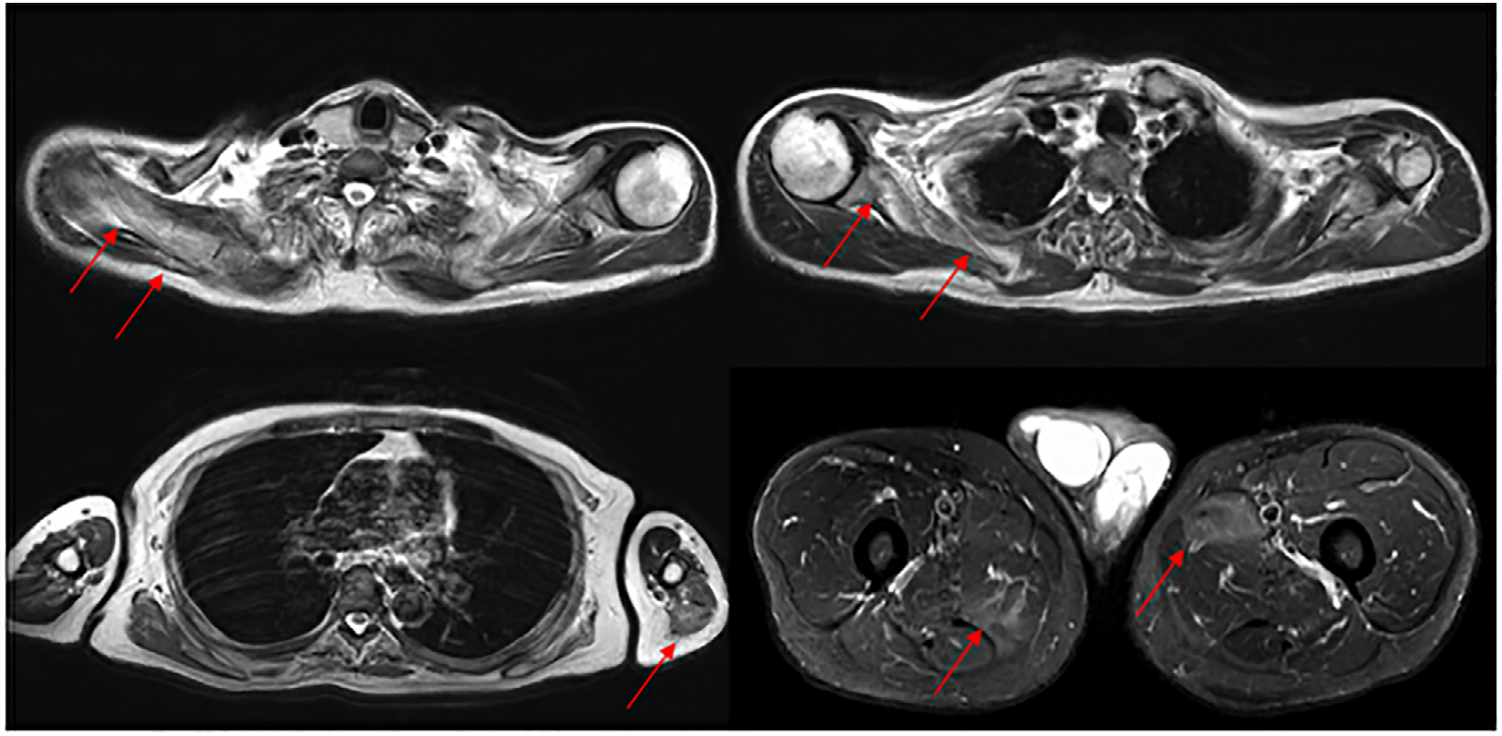
Magnetic resonance imaging (MRI) showed hyperintense areas on STIR images in the right supraspinatus muscle, right subscapularis muscle, left triceps brachii, bilateral obturator externus muscles, right adductor magnus, and left adductor longus. Red arrows indicate hyperintense areas.

**FIGURE 2:**
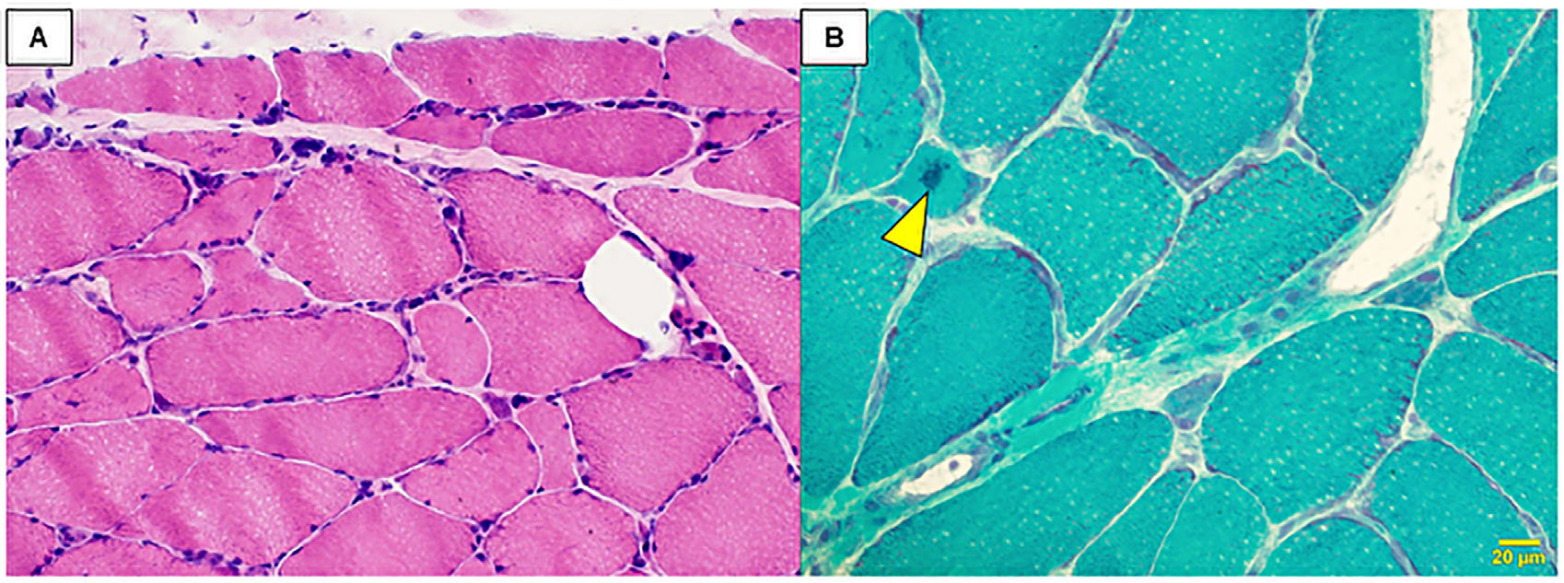
Histopathological finding of muscle biopsy. (A) Hematoxylin and eosin (HE) stain. The findings demonstrated no apparent muscle atrophy and no histological evidence of lymphocytic infiltration or vasculitis. (B) Gomori trichrome stain. Yellow arrows indicate nemaline rods.

Magnetic resonance imaging (MRI) demonstrated hyperintense signals on STIR and T2‐weighted images in the right supraspinatus, right subscapularis, and left triceps brachii muscles. Additional STIR hyperintensity was observed in the bilateral obturator externus, right adductor magnus, and left adductor longus muscles (Figure 1). A muscle biopsy was performed from the left quadriceps femoris. Histopathological examination showed no muscle fiber atrophy, lymphocytic infiltration, or vasculitis, while the presence of a few nemaline bodies was suggested on Gomori trichrome staining (Figure 2).

Laboratory evaluation showed elevated creatine kinase and myoglobin levels, while dermatomyositis‐specific antibodies and anti–acetylcholine receptor antibodies were negative (Table 1). IgG‐κ type M‐protein was detected by serum immunofixation electrophoresis, and the patient was subsequently referred to the Department of Hematology. Bone marrow examination revealed 14.4% clonal plasma cells, without high‐risk cytogenetic abnormalities (*t*(4;14), *t*(14;16), del(17p)). Given that clonal plasma cells accounted for > 10% of bone marrow cells, SLONM associated with smoldering multiple myeloma, rather than MGUS, was strongly suspected.

**TABLE 1:** Laboratory data on admission.

Complete blood count		Biochemistry					
WBC	11,100 /µL	TP	7.2 g/dL	γ‐GTP	20 U/L	IgG	2073 mg/dL
Neut	82.6％	Alb	3.6 g/dL	ALP	254 U/L	IgA	161 mg/dL
Lymp	13.7％	Na	139 mEq/L	CK	498 U/L	IgM	41 mg/dL
Mono	2.8％	K	3.5 mEq/L	ALD	9.2 U/L	Free light chain	
RBC	426 × 10^4^/µL	Cl	103 mEq/L	Myoglobin	130.7 ng/mL	Kappa	681 mg/L
Hgb	13.8 g/dL	Ca	9.1 mg /dL	TG	378 mg/dL	Lambda	15 mg/L
PLT	36.5 × 10^4^/µL	BUN	8.5 mg/dL	HDL‐C	33 mg/dL	κ/λ ratio	45.37
		Crea	0.34 mg/dL	LDL‐C	147 mg/dL	Anti–Jo‐1 antibody	(−)
Coagulation		T‐Bil	0.3 mg/dL	HbA1c	5.7%	Anti–Ach‐R antibody	(−)
PT (s)	10.7	AST	33 U/L	CRP	0.45 mg/dL	Anti–TIF1‐γantibody	(−)
APTT (s)	30.9	ALT	25 U/L			Anti–Mi2 antibody	(−)
D‐dimer	1.0 µg/mL	LD	239 U/L			Anti–MDA5 antibody	(−)

**FIGURE 3:**
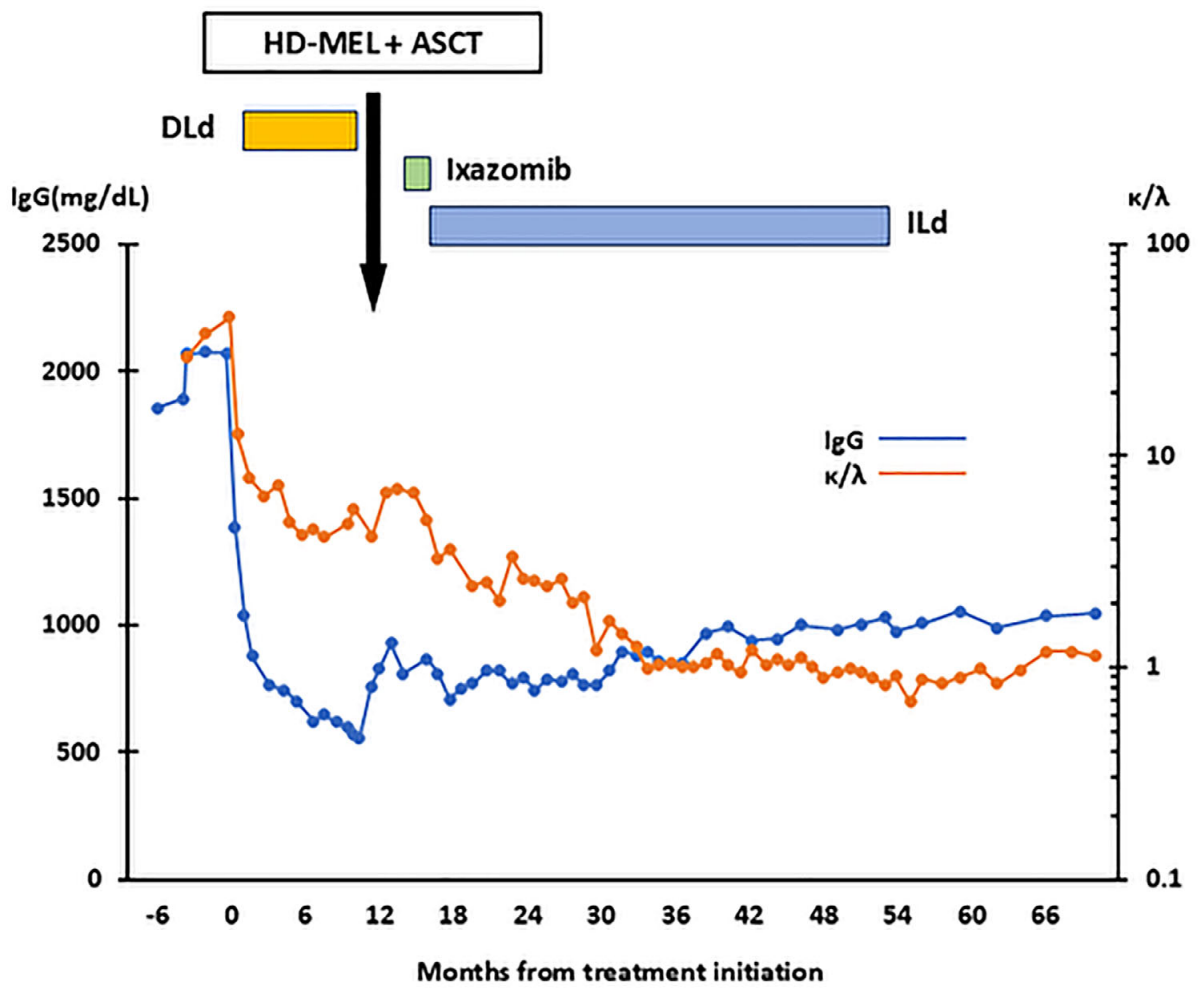
Clinical course and treatment of the patient. ASCT, autologous stem cell transplantation; DLd, daratumumab lenalidomide dexamethasone; HD‐MEL, High‐dose melphalan; ILd, ixazomib lenalidomide dexamethasone; *κ*/*λ*, serum free kappa chain to free lambda chain ratio.

Following the diagnosis, ASCT, which is supported by evidence for SLONM, was considered. However, given the patient's low PS, treatment was initiated with DLd therapy, consistent with standard therapy for transplant‐in‐eligible multiple myeloma. After 11 cycles of DLd, a hematologic partial response (PR) was achieved, accompanied by improvements in PS and muscle strength and a decrease in serum creatine kinase levels to approximately 300 U/L. Peripheral blood stem cell harvesting was subsequently performed, and 2 × 10^6^ /kg CD34^+^ cells were collected. Considering improvement in PS and un‐achievement of a hematological complete response (CR) of the patient, high‐dose melphalan conditioning followed by ASCT was performed, with infusion of 2 × 10^6^/kg CD34^+^ cells. The hematologic response following ASCT remained PR. Two months posttransplantation, ixazomib maintenance therapy was initiated. After three cycles, treatment was escalated to ILd (ixazomib, lenalidomide, dexamethasone) therapy due to remaining IgG‐κ type M‐protein. After 3.5 years from ILd initiation, stringent CR (sCR) and MRD negativity of 10^−5^ by next‐generation multi‐color flow cytometry was achieved. In accordance with the patient's preference, no additional chemotherapy was administered, and the patient has remained progression‐free (Figure 3).

## 3 Discussion

SLONM is a rare neuromuscular disorder characterized by progressive muscle weakness and intrafiber nemaline rod formation. Although its etiology remains unclear, approximately half of cases are associated with MGUS [5, 6]. Associations with HIV infection and autoimmune diseases (systemic lupus erythematosus, Sjögren's syndrome, myasthenia gravis) have also been reported [7, 8, 9], suggesting an immune‐mediated pathogenesis.

Muscle weakness of SLONM typically progresses subacutely and is proximal and symmetrical. These manifestations are more frequent and severe in SLONM with MGUS (SLONM‐MGUS) than in non‐MGUS cases, and fatal outcomes have been reported [3, 5, 6]. SLONM‐MGUS is clinically distinct from multiple myeloma, being characterized by predominant myopathy‐related manifestations, the general absence of myeloma‐related organ dysfunction, and an almost exclusive association with IgG‐type M‐protein [6]. SLONM‐MGUS should also be distinguished from monoclonal gammopathy of neurological significance (MGNS), in which the monoclonal protein is associated primarily with peripheral nerve dysfunction, typically manifesting as sensory or sensorimotor neuropathy. In contrast, SLONM‐MGUS predominantly affects skeletal muscle, resulting in proximal and axial muscle weakness with characteristic nemaline bodies on muscle biopsy [6]. In the present case, adult‐onset proximal and truncal muscle weakness together with MRI abnormalities in symptomatic muscles suggests an acquired myopathy. The presence of monoclonal gammopathy raised suspicion for SLONM, and muscle biopsy supported the diagnosis by demonstrating nemaline bodies on Gomori trichrome staining.

Early diagnosis was possible in this case because nemaline bodies were identified on the initial biopsy. However, prior studies have reported that in nearly half of patients, fewer than 10% of muscle fibers contain nemaline rods, and some cases require electron microscopy for diagnosis because light microscopy findings are inconclusive [3, 5]. Therefore, repeated muscle biopsies should be considered when monoclonal gammopathy coexists with characteristic proximal muscle weakness.

There is no established standard treatment for SLONM. Reported therapeutic approaches include corticosteroids, intravenous immunoglobulin, and immunosuppressive agents such as azathioprine, cyclosporine, and methotrexate. In cases of SLONM‐MGUS, additional treatment strategies, including chemotherapy and ASCT, have also been reported. SLONM‐MGUS appears to be more treatment‐resistant and is associated with higher mortality and poorer functional outcomes compared with SLONM without MGUS [5]. Previous studies have shown superior neurological and survival outcomes in patients treated with chemotherapy and ASCT compared to immunosuppressive therapy alone, with outcomes correlating with reductions in M‐protein levels [10, 11]. These findings suggest that eradication of the underlying plasma cell clone and achievement of hematologic remission may be critical for effective treatment of SLONM‐MGUS. Although the efficacy of ASCT has been reported, the optimal timing of stem cell collection and transplantation remain unclear. Recently, several cases treated with daratumumab have demonstrated favorable outcomes [12, 13], suggesting that daratumumab‐based regimens may represent an effective therapeutic option.

In the present case, DLd therapy was initiated in consideration of the patient's poor PS. Daratumumab was added to lenalidomide and dexamethasone with the aim of achieving a rapid and deep hematologic response, based on the favorable efficacy of the DLd therapy demonstrated in the MAIA trial [14]. DLd therapy resulted in a reduction in the M‐protein level accompanied by improvement in muscle strength. Subsequently, high‐dose melphalan followed by ASCT and ixazomib‐based posttransplant therapy led to sCR and MRD negativity. After achieving MRD negativity, the patient has remained free from relapse and has achieved long‐term survival. However, given the single‐case nature of this report, the efficacy of this treatment strategy cannot be generalized, and further accumulation of cases and additional studies are needed to establish its clinical benefit in SLONM‐MGUS. Nevertheless, these findings suggest that daratumumab‐based induction followed by ASCT may represent an effective treatment option for SLONM‐MGUS. Moreover, like multiple myeloma, achieving MRD negativity may contribute to long‐term survival in patients with SLONM‐MGUS.

## Author Contributions

K.M., Y.K., and M.N. reviewed clinical data and wrote the paper. K.M., Y.K., M.N., N.N., M.U, and J.Y. contributed to patient care and data collection. All authors approved the final version of the manuscript.

## Funding

The authors have nothing to report.

## Ethics Statement

The authors have nothing to report.

## Consent

Informed consent was obtained from the patient for publication of this manuscript.

## Conflicts of Interest

Yawara Kawano has received honoraria from Janssen Pharmaceuticals Inc, Bristol Myers Squibb Co., Ono Pharmaceutical, Takeda Pharmaceutical Co. Ltd., Sanofi, Pfizer, and Sebia outside the submitted work. Nao Nishimura has received honoraria from Bristol Myers Squibb Co. and Sanofi outside the submitted work. The other authors declare no conflicts of interest.

## Data Availability

The data related to this report will be made available from the corresponding author upon reasonable request.
